# Supplementary material for: CDDO-Me Abrogates Aberrant Mitochondrial Elongation in Clasmatodendritic Degeneration by Regulating NF-κB-PDI-Mediated S-Nitrosylation of DRP1
Source: Int J Mol Sci. 2023 Mar 20;24(6):5875. doi: 10.3390/ijms24065875 (PMC10053800; doi:10.3390/ijms24065875)
Supplement: Supplementary file 1 [file ijms-24-05875-s001.zip › ijms-2217732-supplementary.pdf]

Supplementary information

CDDO-Me Abrogates Aberrant Mitochondrial Elongation in Clasmatodendritic Degeneration by Regulating NF-κB-PDI-Mediated S-Nitrosylation of DRP1

Duk-Shin Lee [1,2], Tae-Hyun Kim [1,2], Hana Park [1,2] and Ji-Eun Kim [1,2,*]

1 Department of Anatomy and Neurobiology, College of Medicine, Hallym University, Chuncheon 24252, Republic of Korea

2 Institute of Epilepsy Research, College of Medicine, Hallym University, Chuncheon 24252, Republic of Korea

* Correspondence: jieunkim@hallym.ac.kr; Tel.: +82-33-248-2522; Fax: +82-33-248-2525

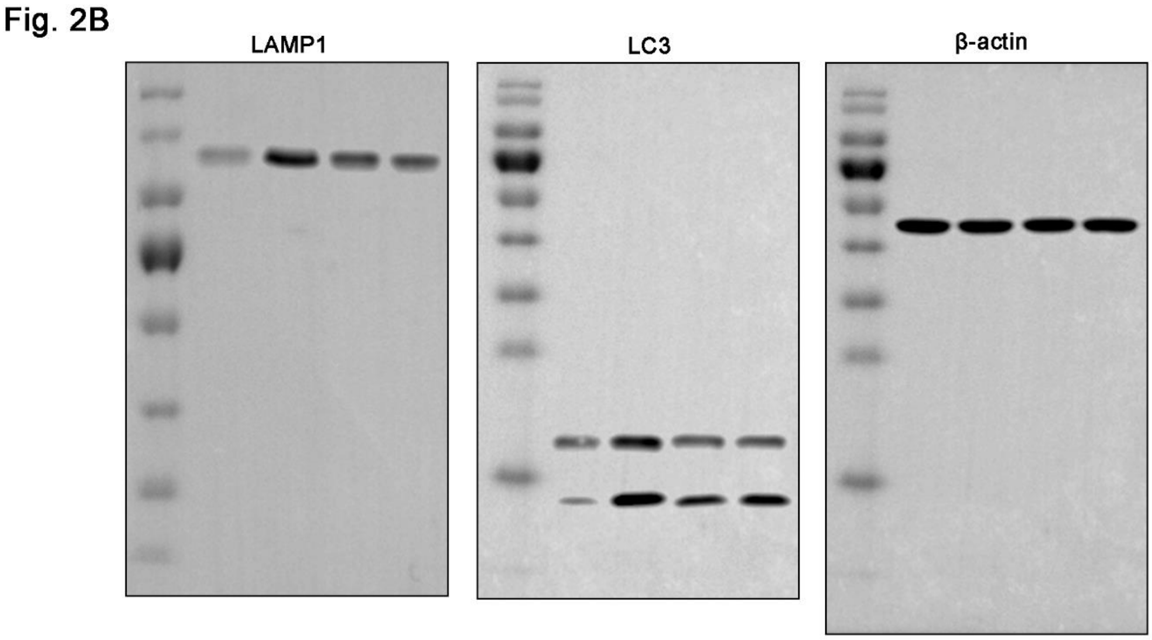

Supplementary Figure S1. Full-length gel images of Western blot data in Figure 2B.

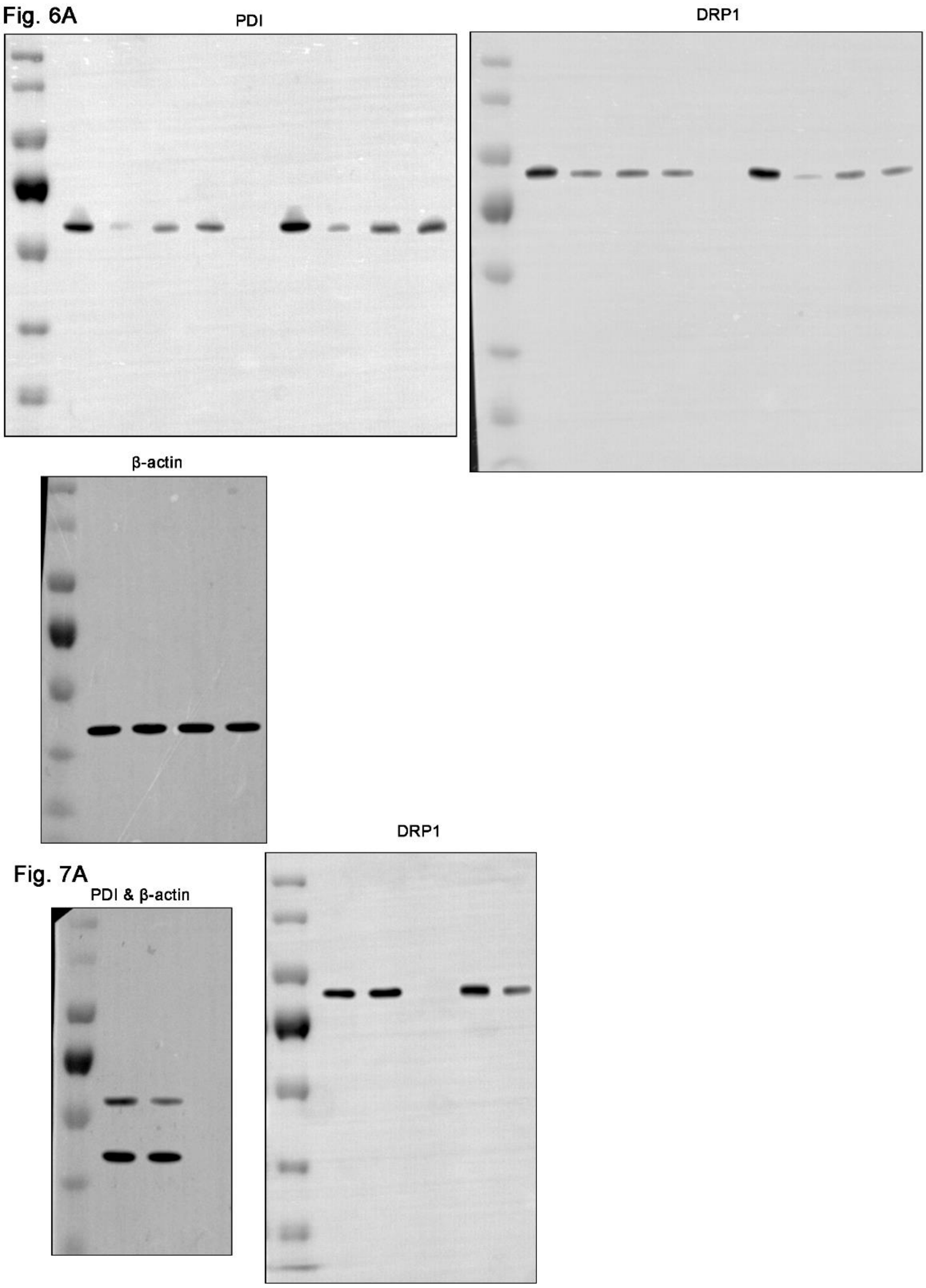

Supplementary Figure S2. Full-length gel images of Western blot data in Figures 6A and 7A.
